# Imagining pandemics now, and then: a century of medical failure

**DOI:** 10.1098/rsfs.2021.0029

**Published:** 2021-10-12

**Authors:** Mark Honigsbaum

**Affiliations:** Department of Journalism, City University of London, London, UK

**Keywords:** medical policy, epidemiology, quarantines, pandemics, coronavirus, influenza

## Abstract

Ever since the devastating 1918–1919 influenza pandemic, policy makers have employed mathematical models to predict the course of epidemics and pandemics in an effort to mitigate their worst impacts. But while Britain has long been a pioneer of predictive epidemiology and disease modellers occupied influential positions on key committees that advised the government on its response to the coronavirus pandemic, as in 1918 Britain mounted one of the least effective responses to Covid-19 of any country in the world. Arguing that this ‘failure of expertise’ was the result of medical and political complacency and over-reliance on disease models predicated on influenza, this paper uses the lens of medical history to show how medical attitudes to Covid-19 mirrored those of the English medical profession in 1918. Rather than putting our faith in preventive medicine and statistical technologies to predict the course of epidemics and dictate suppressive measures in future, I argue we need to cultivate more profound forms of imaginative engagement with infectious disease outbreaks that take account of the long history of quarantines and the lived experiences of pandemics. A useful starting point would be to recognize that while measures such as the R° may be useful for calculating the reproductive rate of a virus, they can never capture the full risks of pandemics or their social complexity.

## 1.

Pandemics have a way of upsetting medical and scientific orthodoxies. A little over 100 years ago a novel virus emerged from an unknown animal reservoir and silently seeded itself in settlements around the world. Then, in the closing months of the First World War, it suddenly began sickening people in Spain and other parts of the Iberian Peninsula.

As with the novel coronavirus, SARS-CoV-2, which emerged without warning in Wuhan, China, in December 2019, sparking the pandemic of Covid-19, at first few medical commentators expressed concern about ‘Spanish influenza’. On learning that the Spanish king, Alfonso XIII, was ill and that similar outbreaks were being reported in England, the *British Medical Journal* condemned the newspaper despatches from Madrid as ‘alarmist’ and suggested the culprit was most likely a type of ‘gastrointestinal influenza’ [1].

Most English medical commentators concurred. Influenza was a regular seasonal visitor to the British Isles and in the previous 1847–1848 and 1889–1892 pandemics clinicians had observed a wide range of gastric, catarrhal and nervous symptoms. Furthermore, English physicians tended to be suspicious of the term influenza, regarding it as vague Italian word for epidemic catarrh [2].^1^ The result was that when in the early summer of 1918 Spanish flu reached the British Isles, medical experts made light of the danger. Nor, when the influenza returned in September in what appeared to be more virulent from, were preparations laid for a pandemic [3].^2^ Instead, English medical authorities put their faith in preventive measures, such as home isolation, the ventilation of sick rooms, gargling with saline water and handkerchiefs to ‘intercept drops of mucus' [4, p. 90].

As Sir Arthur Newsholme, the head of the Local Government Board, and then England's highest authority on medical matters, informed a summit meeting on influenza at the Royal Society of Medicine (RSM) on 13 November, while quarantines and border controls might be effective against cholera and plague, influenza travelled too rapidly and was too extensively diffused. ‘I know of no public health measures that can resist the progress of pandemic influenza’, he stated [5].

Newsholme was not alone in deprecating more forceful preventive measures. ‘With elementary precautions influenza is no more likely to spread than typhoid’, one English hospital doctor informed his colleagues [6]. ‘When epidemics occur, death always happens’, stated the *British Medical Journal*.^3^ But perhaps the most consistent medical advice was to guard against hysteria and try to keep the epidemic in perspective. ‘A stout heart is a great safeguard these days', *The Times'*s medical correspondent advised readers. ‘Fear is certainly the mother of infection. To go about expecting influenza is to invite it’.^4^

It is sobering to read these words today in the light of the experience of Covid-19, a pandemic that, at time of writing, has seen the deaths of 152 000 Britons, the highest number of any country in Europe, and more than four million people worldwide [7]. By contrast, some 228 000 Britons perished in the 1918–1919 influenza pandemic and in excess of 50 million globally [8]. Of course, it is difficult to draw comparisons between Britain's responses to Covid-19 and the 1918–1919 Spanish influenza, a pandemic that coincided with a world war and when there was no such thing as a national health service or intensive care units to treat patients with severe pulmonary infections. Another key difference is that in 1918 disease surveillance was rudimentary and there were no systems such as PROMED or the World Health Organization's Global Outbreak and Response Network to alert governments to trans-national disease threats and mobilize international scientific knowledge and expertise. Nor were there triaging systems and international regulatory mechanisms, such as the 2005 International Health Regulations, for assessing the threat posed by novel pathogens and coordinating supra-national emergency responses. However, one clear parallel is the way that in early 2020, as in 1918, medical professionals, public health administrators and politicians deprecated the severity of the outbreak and, rather than screen travellers at the border and introduce community testing and rigorous contact tracing and quarantines, advised individuals with symptoms of coronavirus infection to self-isolate at home.

Why this was the case will keep historians and committees of inquiry occupied for years. Certainly, it seems paradoxical that Britain should have performed so badly given its concentration of scientific and technical expertise and its detailed contingency planning for a pandemic—planning that had been rated the second best in the world after the USA [9]. Many theories have been advanced for the UK's poor performance, from the under-investment in healthcare under successive Conservative governments, to the distraction of Brexit, to political complacency and the desire to protect Britain's position as a leading trading nation by keeping the border open as long as possible. These factors no doubt all contributed to a greater or lesser extent. However, my focus in this paper is on the role of science—and epidemiology in particular—in the British government's sub-optimal response to the pandemic.

Modelling their response to SARS-CoV-2 on pandemic influenza, some scientists advising politicians appeared to countenance a policy of allowing the coronavirus to spread through the population in order to generate ‘herd immunity’, a term borrowed from vaccinology [10,11].^5^ It was only in mid-March when new projections from the Imperial College modeller and SAGE member, Neil Ferguson, showed the outbreak growing faster than anticipated, and he shared a model estimating that without social distancing and other suppressive measures there could be as many as 250 000 UK deaths, that on 23 March 2020 the government instituted a national lockdown in an attempt to suppress transmission [12]. Richard Horton, the editor of *The Lancet*, has described Britain's failure to institute tougher suppressive measures earlier as ‘the greatest science policy failure for a generation’ [13].

No doubt differences between Britain and China's political systems—and social and cultural differences between European and Southeast Asian populations—also contributed to this failure: for instance, people in Hong Kong, Taiwan and other parts of Southeast Asia were used to quarantines and other suppressive measures thanks to their experience of SARS and bird flu, and Asian public health experts also tended to be distrustful of data coming out of China. Nor were British politicians obliged to follow the advice from epidemiological modellers on SAGE and other advisory groups. However, this failure can also be seen as due, in part, to the over-reliance on mathematical models predicated on influenza to predict and ‘mitigate’ the spread of the coronavirus. While poor data from China in the initial weeks of the outbreak undoubtedly contributed to the unreliability of disease modelling, the shortcomings of the models were exacerbated by epidemiologists' slowness to fully appreciate the role of quarantines in mitigating the impacts of previous epidemics and the public's willingness to restrict their social contacts when faced with an unknown and potentially deadly pathogen. Such insights were well known to medical historians and had been extensively documented in the voluminous literature on plague, cholera, Ebola and SARS but, essentially, had to be rediscovered all over again during the pandemic of Covid-19.

Moreover, medical historians and other students of pandemics, including epidemiologists, often have a gut instinct about the extent of the threat posed by an outbreak of an emerging infectious disease. Sometimes, these instincts can be wrong, as occurred in May 2014 when Pierre Rollin, a viral haemorrhagic fever expert at the Centers for Disease Control and Prevention with experience of ten previous Ebola outbreaks in Africa, informed the *New York Times* that the outbreak in southeastern Guinea ‘was close to over’—the epidemic would subsequently spread to five countries in West Africa and account for more than 11 000 deaths [14]. But just as often these instincts are right: for instance, in January 2020 John Edmunds, professor of epidemiology at the London School of Hygiene and Tropical Medicine and a member of SAGE, reportedly informed a scientific colleague that the coronavirus outbreak was ‘going to be like 1918, a terrible scourge on humanity’ [15].

I am not suggesting that such instincts are a substitute for robust data or should take the place of mathematical modelling; merely that any analysis of the pandemic potential of a novel pathogen, such as SARS-CoV-2, and the management of the resulting crisis should also allow for more imaginative forms of engagement with people's lived experiences of pandemics and the history of quarantines. In brief, I wish to direct attention to the question, what is gained and what is lost when we rely on mathematical technologies to, as Anderson puts it, ‘model our way out of crisis'? [16].

Such an analysis should begin by acknowledging that epidemiology is a statistical abstraction. While measures like R° may be useful for calculating the reproductive rate of a virus and telling us when we need to lock down hard and when we can begin to ease restrictions, it can only ever be an approximation of reality. Like other mathematical concepts employed by epidemiologists, the R° can never capture the full complexity of pandemics and what epidemiologist Gideon Meyerowitz-Katz calls the ‘tangle[d] web of interconnectivity that we call society’ [17].

Moreover, such stochastic models are only as good as the data that go into them and the behavioural assumptions underpinning them. The biggest unknown in the initial weeks and months of the outbreak was the extent to which transmission was being driven by asymptomatic and/or pre-symptomatic carriers. In the absence of evidence from China to the contrary, many experts accepted the assurances of Chinese officials that laboratory tests had detected the majority of infections. For example, on his return from a fact-finding mission to China in February 2020, Bruce Aylward, co-lead of the WHO-China Joint Mission onCoronavirus, informed the *New York Times*: ‘There is no evidence that we're seeing only the tip of a grand iceberg, with nine-tenths of it made up of hidden zombies shedding virus. What we're seeing is a pyramid: most of it is above ground’ [18]. In fact, based on data from Singapore, Hong Kong and China, it is now thought that between a third and 60% of people infected with the virus may be asymptomatic [19]. Moreover, unlike influenza, which has a two- to four-day incubation period, on average it takes 5–6 days and, in some cases as long as 14 days, for a person infected with SARS-CoV-2 to develop symptoms. That gives authorities a one- to two-week window in which to test, trace and isolate asymptomatic but potentially infectious individuals and suppress coronavirus infection chains before they can spiral out of control. SARS-CoV-2 is also at least twice as infectious as pandemic influenza, with a reproductive ratio or R°, without interventions, of 1–4.9. That compares to an R° of 1.8 for Spanish influenza and an R° of 1.46 for the 2009 H1N1 swine influenza [20].

Another highly questionable assumption was that social distancing measures should be time-limited in case of ‘behavioural fatigue’ if the government locked down too soon. This issue had first been raised by England's Chief Medical Officer Chris Whitty at a Downing Street coronavirus briefing in early March and subsequently repeated by British prime minister, Boris Johnson, and the UK's chief scientific adviser, Sir Patrick Vallance [21].^6^ The policy is thought to have originated either with behavioural psychologists on the Scientific Pandemic Influenza Group on Behaviours (SPI-B), or the Downing Street Behavioural Insights Team, also known as the ‘Nudge Unit’. However, members of both bodies have denied giving this advice and on 13 March SAGE explicitly advised politicians not to delay any appropriate measures owing to concerns over ‘difficulty maintaining behaviours’ [21]. Jeremy Farrar, the director of the Wellcome Trust and a prominent member of SAGE, has since described the concept of behavioural fatigue as ‘a peripheral idea promoted beyond any merit or science’ [22, p. 136].

But perhaps the biggest error was the UK's failure to introduce strict border controls and the government's decision in March to abandon community test and trace. Asian countries such as Taiwan, South Korea, Singapore and Hong Kong introduced strict border controls and quarantines and aggressively deployed test, trace and isolate within days of learning of the outbreak in Wuhan [23]. Similarly, Italy banned flights from China on 31 January and Germany restricted travel to and from Wuhan in February. However, the UK's then Health Secretary Matt Hancock declined to impose equivalent controls, citing advice from Whitty that travel restrictions would only delay the spread of the disease ‘by a matter of days’ [24]. This was followed on 13 March by Whitty's announcement that the UK was suspending community surveillance and that henceforth only hospitalized patients would be tested for coronavirus. It subsequently emerged that Imperial College had stopped modelling strategies based on widespread testing after 28 January, when Public Health England informed Ferguson that it lacked sufficient capability [25]. However, the decision also appears to have reflected an assumption in March by SAGE advisors that there was already widespread community transmission in Britain and that, based on the experience of influenza, there was little that could be done to inhibit spread of the coronavirus.^7^

All of which begs the question, what prompted the sudden shift in policy and the UK government's decision to lockdown hard on March 23? After all, this was not the first time that authorities had employed social distancing measures in an attempt to ‘flatten the curve’ of an epidemic. In 2007, a study of non-pharmaceutical interventions in the USA during the Spanish flu pandemic showed that cities such as St Louis where in 1918 authorities banned public gatherings and employed other social distancing measures, succeeded in reducing the peak of the epidemic and were also able to quickly restore economic activity. By contrast, cities such as Philadelphia that permitted Liberty Loan parades and other large public gatherings to go ahead, suffered higher mortality from influenza and worse economic impacts too [26].

Similarly, in England, despite the absence of directives from the LGB, in October 1918 James Niven, the Medical Officer of Health for Manchester, posted prominent warnings urging Mancunians to avoid public gatherings for at least ten days from the commencement of an influenza attack. Although Niven's appeals did not prevent civilians and soldiers thronging Manchester city centre on 11 November 1918, to celebrate the Armistice, just 1715 Mancunians died in the second wave of the pandemic. By contrast, in London, where no warnings were posted about the dangers of public gatherings, there were around 16 000 deaths [4, p. 105; 27]. The abrupt change in mortality between the spring and autumn prompted the epidemiologist and early disease modeller, Major Greenwood, to compare the increase in cases between the first and second waves of the Russian influenza pandemic in 1890–1891 with the first two waves of Spanish influenza. In both cases, Greenwood was able to show that the waves had exhibited near symmetrical rises and falls, the only difference being that in 1891 the follow-on wave of Russian flu had coincided with the spring, whereas in 1918 the secondary wave coincided with the autumn [28]. Newsholme, however, was unimpressed with Greenwood's new statistical methods, telling the summit meeting at the RSM that no one could have anticipated the return of influenza in the autumn and bristling at suggestions that ‘more could have been done to avert the present pandemic’ [5].

Fast forward 102 years, however, and by February 2020 China had demonstrated to the world just how effective the lockdowns of Wuhan and other large cities in Hubei province could be, especially when combined with aggressive contact tracing and case management. ‘Within Hubei, the implementation of control measures (including social distancing) has reduced the community force of infection, resulting in the progressively lower incident reported case counts’, reported the WHO Joint Mission in February.^8^ Populations in other WHO member states, including, by implication, those of advanced Western democracies, should get ready to embrace ‘more stringent’ social distancing, the WHO team concluded [5, p. 22].

That report would mark a pivotal turning point in the British response, persuading Steve Riley, a member of the SPI-M modelling group, to revisit key behavioural assumptions about the public's tolerance of lockdowns and other social distancing measures. In particular, it would expose the fallacy at the heart of the herd immunity strategy as Riley concluded that were the virus to be allowed to spread unimpeded the British public would spontaneously adopt social distancing out of fear as infections rocketed and intensive care units filled to capacity [22, pp. 108–110]. Such fear-induced behaviours had been observed in Hong Kong following the quarantining of apartment blocks during the 2003 SARS outbreak. Similar spontaneous behaviour changes had been observed in Liberia and Sierra Leone during the 2014–16 Ebola outbreak, as well as during outbreaks of plague and cholera in the seventeenth and nineteenth centuries [29]. Moreover, though quarantines frequently provoke resistance, they are a familiar response to outbreaks, one whose logic has changed little since 1377 when Dubrovnik passed an ordinance requiring travellers from plague-infested areas to isolate for a month on an island beyond the city's limits. But curiously, while countries like Taiwan, Singapore and Hong Kong were quick to close their borders to travellers from China suspected of harbouring the coronavirus and adopt quarantines and social distancing, SAGE decided such measures would be of little utility and prove counterproductive over the long term. ‘Measures which are too effective merely push all transmission to the period after they are lifted, giving a delay but no substantial reduction in either peak incidence or overall attack rate,’ stated a SAGE report on non-pharmaceutical interventions on 25 February. [‘Potential effect of non-pharmaceutical interventions on a COVID-19 epidemic’, SAGE, 25 February 2020. Available at: https://www.gov.uk/government/collections/sage-meetings-february-2020#meeting-10,-25-february-2020, accessed 19 July 2021.] ‘There is some evidence that people find quarantining hard the longer it goes on… There is no comparable evidence for social distancing measures, but experience suggests it is harder to comply with a challenging behaviour over a long period than over a short period’, read another on 13 March. [“Fifteenth SAGE meeting on Wuhan Coronavirus”, 13 March 2020. Available at: https://www.gov.uk/government/publications/sage-minutes-coronavirus-covid-19-response-13-march-2020, accessed 19 July 2020.] Little wonder that Farrar recalled that, ‘At the end of February 2020, there was almost disbelief, including from me, that (widescale social distancing and lockdowns) was possible.’ [Farrar, Spike, p. 95] This was a fundamental imaginative failure. As Gabriel Leung, a leading Hong Kong infectious disease expert, argued at a presentation at the London School of Hygiene and Medicine (LSHTM) on 27 February, the fact that Hong Kong's population had gone along with quarantines and social distancing measures, despite the deep distrust of government that followed the social unrest in the former crown colony in 2019, suggested that, ‘If Hong Kong can do it anybody can’ [Gabriel Leung, “Nowcasting COVID-19 for Public Health Control: Learning from the Chinese Experience for Global Preparedness”, London School of Hygiene and Tropical Medicine, 27 February 2020. Available at: https://lshtm.cloud.panopto.eu/Panopto/Pages/Viewer.aspx?id=83ba0783-b1ce-4053-aaa5-ab6600da76d8 (accessed 19 July 2020)].

The other key development was the coronavirus outbreak in Lombardy, in northern Italy, on 21 February. As the Italian Prime Minister Giuseppe Conte ordered an immediate lockdown to prevent hospitals in the province being overwhelmed, it became clear that the virus had been spreading stealthily under the radar in Northern Europe for some time and that projections of the number of UK cases had been grossly underestimated. Drawing on models predicated on the transmission of avian influenza, on 16 March, Ferguson recalculated the impact of mitigation and realized that long before herd immunity could be reached, the National Health Service would be overwhelmed [30]. This was not only because a larger proportion of the UK population had most likely been infected than previous models had assumed but because modelling by age showed that a third of those aged 70 to 79 were likely to require hospitalization and 5.1% might die (previous models had estimated the attack rate in this age group as 16.6% with an infection fatality rate of 4.3%). At best, Ferguson calculated, a combination of all the contemplated mitigation strategies would restrict UK deaths to 250 000. The worst-case scenario, however, was 550 000 deaths. ‘Prior to this epidemic, I don't think suppression of a respiratory disease was really an option in the modern world (even though US cities tried it in 1918)’, Ferguson informed another researcher at University College London. ‘China showed it was possible, though it was late February before it was clear it had worked’ [25].

Unfortunately, Ferguson's bleak assessment came too late to prevent the Cheltenham Gold Cup going ahead. On 13 March, 125 000 people converged on the Midlands for four days of drinking and raucous cheering. Four days later, on 17 March, SAGE called for extra social distancing ‘as soon as possible’ but it was not until 23 March that Boris Johnson finally instructed Britons that they ‘must stay at home’ in order to protect the NHS and ‘save lives’.^9^ Ferguson would later acknowledge that ‘mitigation was synonymous with the acquisition of herd immunity’ and that some 20 000 deaths could have been avoided by locking down a week earlier [22, p. 97] (see also [31]).

In 1918, disease modelling was in its infancy and, despite Greenwood's efforts to warn Newsholme of a second wave of Spanish influenza in the autumn, epidemiologists were not at the heart of policy making. Moreover, unlike cholera and plague, influenza was not a notifiable disease in 1918 and infectious disease experts were confident that simple preventive measures were sufficient to check its spread. Nevertheless, revisiting Britain's nonchalant response to the 1918–1919 influenza pandemic, Sandra Tomkins reached a similar conclusion to Horton in 2020, finding the English medical profession guilty of a ‘failure of expertise’. She concluded: ‘The paradox emerges that Britain, with one of the most sophisticated public health machineries of the period, mounted one of the least effective responses to the epidemic’ [6, p. 437]. This paper has argued that Britain's medical establishment suffered a similar failure of expertise in 2020. Unlike in 1918, however, this failure was not due so much to misplaced faith in the power of preventive medicine as over-reliance on epidemiology to model the coronavirus pandemic and anticipate the measures needed to suppress it. The result was that rather than locking down hard in early March, the government appeared to countenance a policy of permitting the coronavirus to spread through the population in an effort to generate herd immunity. Although this policy was never explicitly presented to or endorsed by SAGE, herd immunity was implicit in the mitigation scenarios modelled by Imperial and other groups. As Ferguson told Farrar: ‘It [mitigation] meant we weren't trying to stop something’ [22, p. 97]. The irony is that in 1918 Greenwood and other early pioneers of predictive epidemiology also thought it ought to be possible to anticipate the occurrence of epidemics and mitigate their impacts by extrapolating from epidemiological patterns observed in the past. ‘Some who have not sufficiently attended to the matter have objected that the conception is fatalist, that it amounts to postulating of epidemic diseases an inevitableness which deprives sanitary administration of any hope of basis of success’, Greenwood observed in the Ministry of Health's *Report on the Pandemic.* ‘The very reverse of this is the proper inference’ [32, p. 193].

Greenwood's words were a not-so-coded dig at Newsholme, who had earlier scoffed at his warnings of a second wave as ‘a foolishly wild guess' [5, p. 12]. However, Greenwood's methods were enthusiastically taken up by George Newman, the Chief Medical Officer at the new Ministry of Health who answered directly to Secretary of State Christopher Addison. In his preface to the report on the pandemic, Newman argued that if a reliable way could be found of predicting epidemics then their ‘ravages may be mitigated, perhaps altogether checked’. The experience of Spanish influenza had underlined ‘the essential solidarity of all mankind in the matter of epidemic sickness', he continued. Measures such as sanitary cordons and quarantines could only delay pandemics, not prevent them. Anticipating the establishment of the Health Division of the League of Nations, Newman argued that what was needed was a ‘supra-national system of preventive medicine’. In the meantime, Newman made influenza a notifiable disease and appointed a standing committee of the medical heads of various Whitehall departments to share ‘intelligence’ on diseases with epidemic and pandemic potential. Despite these innovations, however, Newman was ‘gloomy’ about the prospects for preventive medicine. ‘That we have just passed though one of the great sicknesses of history… is an experience which should dispel any easy optimism of the kind. No instructed epidemiologist can say that the world may not have to endure during the next half century other plagues of the first order of severity’ [33].

Writing in the midst of a severe coronavirus pandemic that few people anticipated, that is a verdict with which most experts, including epidemiologists, ought to concur. Reviewing the history of influenza in 1920, Greenwood blamed the Spanish influenza pandemic on a combination of urban overcrowding and ‘the provision of countless incubators, whether in garrisons, war-time factories or abnormally overcrowded and ill-ventilated means of transport’ [32, p. 190]. But whereas a century ago we might have expected epidemics to be circumscribed by geography, today, thanks to international jet travel and faster rail and shipping connections, a novel pathogen emerging in one part of the world can be anywhere on the globe within a matter of days. Nor, despite the advances in microbiology, immunology, vaccinology and preventive medicine since 1919, are we any closer to being able to predict when influenza, or any other pandemic virus, will emerge or to what extent we can mitigate its impact on human populations. As the late American virologist, Edwin Kilbourne, observed in 2006: ‘We can prepare, but with the realization that no amount of hand-washing, hand wringing, public education or gauze masks will do the trick’ [34]. Nor should we make the mistake again of allowing mathematical models to become a substitute for other, more imaginative forms of engagement with pandemics. Even now, hardly a day goes by without some announcement about the R°, but as useful as this measure may be for plotting the rate of increase of the coronavirus and the effectiveness of vaccines in preventing hospitalisations and deaths, it does not tell us when or how we will be delivered from our pandemic purgatory, much less what we need to do to prevent disaster recurring. As Tedros Ghebreyesus, the WHO's Director General, remarked in June 2019, on the six-month anniversary of the outbreak in Wuhan: ‘None of us could have imagined how our world—and our lives—would be thrown into turmoil by this new virus’.^10^

We cannot allow our imaginations to fail us so catastrophically again.

## Endnotes

1 As *The Times's* medical correspondent put it in 1848 [2].

2 For discussion as to whether there was a change in virulence between the spring and autumn waves of Spanish influenza, see [3].

3 *British Medical Journal,* 9 November 1918.

4 *The Times*, 31 October 1918.

5 In an interview with BBC Radio 4 on 13 March, Sir Patrick Vallance said the UK's strategy was ‘to build up some degree of herd immunity while protecting the most vulnerable’ [10]. The same day, in an interview with BBC Newsnight, Graham Medley, the chair of the Scientific Pandemic Influenza Group on Modelling (SPI-M), advocated a ‘nice, big epidemic’ to generate herd immunity (https://www.bbc.co.uk/programmes/p086hjgc). Although Vallance subsequently sought to distance himself from these remarks and Whitty has denied ever countenancing a herd immunity strategy, senior Conservative politicians interviewed by the Sunday Times Insight team insist that Whitty and Vallance were ‘absolutely focussed on herd immunity’ [11].

6 Speaking at a televised briefing on 9 March, Whitty informed viewers: ‘There is a risk that, if we go too early, people will understandably get fatigued and it will be difficult to sustain this over time’. On 12 March, Whitty added: ‘An important part of the science on this is actually the behavioural science, and what that shows is … that people start off with the best of intentions, but enthusiasm at a certain point starts to flag’ [21].

7 Addendum to fifteenth SAGE meeting on Covid-19, 13 March 2020. https://www.gov.uk/government/publications/sage-minutes-coronavirus-covid-19-response-13-march-2020.

8 Report of the WHO-China Joint Mission on Coronavirus Disease 2019 (COVID-19), p. 10. See https://www.who.int/docs/default-source/coronaviruse/who-china-joint-mission-on-covid-19-final-report.pdf (accessed 14 July 2021).

9 See https://www.gov.uk/government/speeches/pm-address-to-the-nation-on-coronavirus-23-march-2020.

10 https://www.who.int/director-general/speeches/detail/who-director-general-s-opening-remarks-at-the-media-briefing-on-covid-19---29-june-2020.

## Data accessibility

This article has no additional data.
